# Between animal research and animal welfare: Analysing the openness practices of UK Named Veterinary Surgeons

**DOI:** 10.1017/awf.2024.42

**Published:** 2024-09-23

**Authors:** Renelle McGlacken, Pru Hobson-West

**Affiliations:** School of Sociology and Social Policy, University of Nottingham, Nottingham, UK

**Keywords:** Animal research, animal welfare, dirty work, laboratory, openness, veterinarians

## Abstract

The use of animals as scientific models is argued to be crucial for producing new scientific and medical knowledge and clinical treatments. However, animal research continues to raise socio-ethical concerns. In recent years, there has been a push for openness amongst the life science community, with the aim of increasing the transparency of animal research to wider publics. Yet, how this push for openness is experienced by those responsible for the care and welfare of research animals requires further study. This paper draws upon qualitative interviews with Named Veterinary Surgeons (NVS) in the UK and explores how they practise openness, avoid openness, and, at times, challenge the way their role is represented within openness agendas. Overall, this social scientific analysis reveals that the current openness agenda has the potential to create tensions for professionals, as they seek to manage regulatory and public imaginaries of the veterinary identity alongside the animal research controversy. The paper concludes by arguing for a culture of dialogue, where openness includes allowing those with responsibilities for animal welfare to express ambivalence or concern about their own role. Finally, the paper calls for sustained academic work on relations between the veterinary profession and wider society, particularly areas that involve contested practices in which care and harm may coincide.

## Introduction

### Animal welfare and the Named Veterinary Surgeon

Non-human animals are widely used as models in scientific research aimed at producing scientific and medical knowledge, and in the development and testing of new clinical treatments. This use of animals primarily for human benefit raises important ethical and welfare-related questions and has been the subject of sustained debate. These questions include how to ethically assess the use of animals in science and which frameworks and principles are appropriate (see Beauchamp & DeGrazia 2020), but also how to appropriately house, care for, and kill different animal species in laboratories.

In the UK, the law mandates that named individuals have specific responsibilities for laboratory animal welfare (Home Office 2014a). Amongst others, this includes the Named Veterinary Surgeon (NVS) who “*is responsible for, monitors and provides advice on the health, welfare and treatment of animals and should help the establishment licence holder to fulfil his/her responsibilities*” (Home Office 2014b; p 72). The NVS must also sit on the establishment’s ethics committee, known as the Animal Welfare and Ethical Review Body (AWERB), and is positioned as a key actor in fostering institutional ‘cultures of care’ (Brown *et al.* 2018).

In addition, as with all qualified veterinarians, the NVS also has professional responsibilities to “*other veterinary surgeons, to the public and to the Royal College of Veterinary Surgeons*” (*ibid*). In terms of the public, the NVS is often claimed to be the most trusted source of “*balanced information on the use of animals in research*” (Ipsos MORI 2018; p 10). This image may reflect a wider reputation of veterinarians generally as “*among the most trusted professionals in Great Britain*” (Veterinary Record 2015) and “*a leading force for animal health and welfare*” (Institute for Employment Studies [IES] 2019; p 134). Such claims of strong public trust in veterinary professionals may also signify dominant imaginaries of the veterinarian as an “*advocate for animals*” (see McGlacken *et al.* 2023) and someone who “*will cure* [one’s] *animal when it is ill*” (Wilkins 2008; p 1). This apparent high level of public trust in veterinarians could also relate to lingering romantic stereotypes of the James Herriot veterinary figure (Hendrix *et al.* 2006).

However, in the animal research context, veterinarians must navigate the provision of expert care and welfare for animals within practices of (sometimes intentional) harm. Indeed, examining the professional ethical standards underpinning the role of veterinarians working in animal research facilities within the European Union, Kiraga and Dzikowski (2023; p 10) point out that “*The regulations themselves do not indicate to the veterinarian what ethical decisions or what choices to make*” but rather only issue “*directives of conduct*”. Thus, working in such spaces requires veterinarians to make at times difficult ethical decisions. In this way, veterinarians working in animal research and other non-therapeutic contexts might be regarded as challenging “*the wider professional norms of what it means to be a veterinary surgeon in the 21st century*” (Ashall & Hobson-West 2018; p 295). As Wolfensohn, long-time head of veterinary services at the University of Oxford, UK outlined in the journal *Nature*:

“*As a vet surgeon you take an oath when you qualify that says that your constant endeavour will be for the welfare of animals committed to your care, but when you’re dealing with experimental animals, their welfare has the potential to be compromised. That inevitably puts you in a difficult position*” [Smith 2006; p 811].

### Openness and animal research

Yet, what happens to the ‘difficult position’ that NVSs experience at the intersection of care and harm when it is subject to a push for laboratory personnel to be more open about their roles within animal research? In the UK, the *Concordat on Openness on Animal Research* was launched in 2014 by research advocacy organisation, Understanding Animal Research (UAR 2014). In signing up to the concordat, UK research funders, universities, and commercial organisations make commitments to improving transparency, openness, and data-sharing about why, when, and how animals are used in scientific research and testing(UAR 2022). In recent years, several other countries have established similar openness agreements (European Animal Research Association [EARA] 2023), including New Zealand (ANZCCART 2021) and Australia (ANZCCART 2023).

An increase in openness and transparency around scientific animal use is claimed to have multiple benefits. For example, Reed from the Royal Society for the Prevention of Cruelty to Animals (RSPCA) describes it as a “*willingness to communicate meaningful information to others in a spirit of trust in the hope that such openness will bring mutual benefit*” and argues that, in the context of animal research, “*improved openness also can lead to benefits for animals, as third parties*” (Reed 2012; p 251). Openness and transparency have also been linked to cultivating a ‘culture of care’ in research environments, which “*supports staff, animal welfare, open communications, transparency and high quality science*” (Robinson & Kerton 2021; p 270). Others have claimed that the openness agenda could help foster public trust towards animal research (see McLeod & Hobson-West 2015; Muñoz-Tamayo *et al.* 2022). Indeed, a decline in public trust towards the regulation of animal research identified through UK opinion polling (Ipsos MORI 2013) originally helped catalyse the initial establishment of the UK’s Concordat (Jarrett 2016; MacArthur Clark *et al.* 2019). Finally, organisations representing animal research have also advocated a cultural shift from a situation where individuals were afraid to discuss their role, to a point in which this becomes part of the ‘natural discourse’ (van Paridon & Chain 2020).

Whilst such overarching goals of the openness agenda may seem clear, putting openness into practice may prove challenging. With the potential for stigma or taboo around the killing of non-human animals (McCabe & Hamilton 2015; Tallberg & Jordan 2021), professional involvement in scientific uses of animals may be stigmatised (Birke *et al.* 2007; Johnson & Smajdor 2019) or subject to ‘moral taint’, with the latter occurring “*where an occupation is generally regarded as somewhat sinful or of dubious virtue*” (Ashforth & Kreiner 1999; p 415). Such concepts have previously been applied to animal research by sociologist Arluke (1991). Animal research also featured in Ashforth *et al.*’s classic (2007; p 160) study of managers in ‘dirty work’ occupations. Going further, Tallberg and Jordan (2021; p 3) suggest that dirty work involving animals raises particular challenges, given that “*work with animals involves a different set of moral norms [including, at times, killing] – treating animals in ways that would not be sanctioned for a human population*”.

The ‘dirtiness’ of occupational involvement with scientific animal use has an important entwinement with the UK’s history of protest and activism around the issue and the ‘legacy’ (McLeod 2018) of intimidation and violence against those associated with the practice from the 1990s and 2000s (Illman 2005). McLeod (2018; p 62) has written of a ‘continuing tension’ amongst the animal research community in “*seeking out support and trust from the wider public through greater transparency*” whilst also being fearful of ‘dangerous’ publics who “*may put scientists or institutions in jeopardy*”. Given increasing pressures placed upon animal research personnel to be open about their work, they identify “*the importance of research to understand the challenges and experiences of scientists and institutions who are being asked to embrace transparency initiatives*” (*ibid*; p 68).

Although many of the sociological studies of how individuals involved in scientific animal use discuss their work predate the mobilisation of openness agendas, this existing literature remains helpful in identifying the longstanding challenges of openness around professional involvement in animal research. Such empirical studies have shown how animal research personnel may hold concerns that publics will scrutinise their work (Hobson-West 2012) or regard it as discreditable or stigmatised (Arluke 1991; Birke *et al.* 2007; Holmberg & Ideland 2010; Brunt & Weary 2021). Indeed, Ashforth *et al.*’s (2007) interview study of managers of “*stigmatized occupations*” or “*dirty*” professions found that animal research managers engage in several defensive tactics against the stigmatisation of their work. These tactics may involve ‘social buffering’, i.e. “*restricting his or her focus to an inner circle of vetted people*”; “*social comparison*” between those involved in animal research versus animal *testing*; and “*condemning the condemners*”, i.e. criticising the critics and thus dismissing the legitimacy of their critique (*ibid*; pp 160–165). Yet, importantly, Arluke (1991; p 325) notes that whilst stigma or taint may be experienced across roles, animal technicians (those involved in the daily care of animals in research facilities) were more likely to *hide* their involvement in animal research.

More recent scholarship in different regions has also identified differences between animal research staff. For example, Holmberg and Ideland’s (2010; p 362) ethnographic study of openness and secrecy amongst laboratory personnel in Sweden found that animal technicians and veterinarians occupy a particularly difficult dual position in relation to openness, “*representing the public but also afraid of what the public may think of them*”. In examining contributing factors to compassion fatigue amongst laboratory animal personnel in the US and Canada, LaFollette *et al.* (2020; p 8) observe that “*some laboratory animal personnel may have difficulty gaining work-related social support because of the stigmatization of the field*”. Correlating this with higher levels of compassion fatigue, the authors suggest “*encouraging greater openness in talking about research or establishing support groups*” (*ibid*). Relatedly, Brunt and Weary’s (2021; p 8) Canadian study of animal research facility managers found that some described their work as stigmatised, with a number of managers feeling that even the university hospital they work in “*viewed animal research as tainted, with the potential to tarnish the institution’s reputation*”.

So, how do veterinarians involved in animal research experience the push for openness around animal research? As was already highlighted, their role is a complex one. Yet there is relatively less social scientific work focused upon veterinarians in this space. One key exception to this is provided by Brunt and Weary (2023) who, in an article also published in this journal, show that veterinarians working in the Canadian animal research context identify “*institutional fears*” as preventing transparency (p 4), with a majority of respondents calling for a “*unified approach*” which involves “*all universities increasing transparency in the same way at the same time*” (p 5–6). With veterinarians playing a key role in promoting and protecting animal welfare in the research environment, our paper aims to contribute to and extend this social scientific literature by examining how veterinarians working in animal research facilities (in the UK, the ‘Named Veterinary Surgeon’ or ‘NVS’) perceive and experience the push for openness around animal research on an interpersonal level. Based on an analysis of in-depth interviews with UK NVS veterinarians, this paper examines the ways in which Named Veterinarians enact openness, sometimes seek to avoid it, and at times even challenge the representation of their role in the openness agenda.

## Materials and methods

Our study of the role of the NVS is part of a wider programme of interdisciplinary research which focuses on animal research in the UK, the Animal Research Nexus (see Davies *et al.* 2024). Based on qualitative interviews with Named Vets, our empirical phase received ethical approval from the University of Nottingham (approval number: 1800160608) and data collection began in 2018. A semi-structured interview guide (available in Anderson & Hobson-West 2022) was developed and discussed with an expert advisory panel consisting of three Named Veterinarians and trialled during two pilot interviews. The interview guide did not include specific prompts on openness. However, the guide included a section on ‘how others see the NVS’, where interviewees were invited to consider how their role is perceived. This generated interesting reflections on the topic of openness which are analysed in this paper. As is standard practice in qualitative studies of this type, the interview prompts were not read out *verbatim*, but were used as prompts for a wide-ranging discussion.

In terms of recruitment, NVS participants were contacted through snowball sampling which was initiated via personal networks and a call for participants during a specialist conference. Thirty-three NVSs were interviewed in person at locations chosen by the participants, and all were in current employment (full or part-time) as an NVS. Participants were based at universities or commercial organisations, with ranging lengths of experience in the role. All interviewees signed a consent form and received a participant information sheet, with details regarding the project and the use of personal and research data. Interviews were recorded and transcribed by a third party under a confidentiality agreement. Transcripts were anonymised and decontextualised, with all identifiable material regarding names, locations, and organisations removed. Each transcript was assigned a gender-specific pseudonym. Assurances of anonymity may have helped to encourage participation.

The dataset analysed here was subjected to several kinds of constructivist thematic analysis (Braun & Clarke 2006) involving different members of the research team. In the first stage, an inductive approach was adopted, where transcripts were coded line-by-line to explore patterns and themes within the data. This inductive approach was deemed an important step given the understudied nature of the NVS role. Outcomes from this stage are reported elsewhere and focus on career history (Anderson & Hobson-West 2022), ideas of animal advocacy (McGlacken *et al.* 2023), and how animal research is compared with other forms of veterinary work (Anderson & Hobson-West 2023). The current paper is based upon a second stage of analysis. In this phase, we benefited from the data organisation and original coding produced in the first phase, but more deductively investigated the dataset for instances of discussion of animal welfare and openness. This deductive analysis was led by RM; both authors participated in the construction of themes, and in the writing and organisation of this paper. In what follows, we cover three topics: how NVSs practise openness around their role, avoid openness, and challenge the representation of their role within the openness agenda. To conclude, we draw out the implications of this analysis for animal welfare.

## Results and discussion

### Practising openness

The interviews with veterinarians included accounts of the way in which others can perceive their role negatively. One commonly discussed response was to try to educate critics about the care practices, welfare standards, and regulations governing animal research. As Mabel suggests in this extract, this involves a distinction between working for the *animals* versus the *research*:

“*I just worry that people automatically perceive things in a negative light, so I tend to just explain that I look after the animals’ health and welfare and make sure that people aren’t doing anything that would be detrimental to that. So that if there is a problem, I can highlight it and that animal wouldn’t come to any harm, but I still don’t think people really understand what I do* […]” [Mabel].

As Mabel notes, because of the “*negative light*” cast over animal research, assumptions and judgments of the NVS role are countered by emphasising their responsibility to protect the welfare of research animals from the research itself. Similarly, Molly explains that although the culture around animal research generally has changed, they still encounter negative reactions when telling people about their work:

“[…] *animal research has changed. Before I used to lie about what I did all the time and now I don’t feel I have to, but I sometimes am surprised that people’s reactions when I do say what I do. It’s horror really, “How could you do that?”, and people change to you actually, change their attitude, avoid you a little bit. Instead of thinking you’re a vet, this wonderful person that they look up to, you’re like the scum of the earth that they don’t really want to talk to anymore, so I have had that…But I feel much more comfortable in being able to talk about my role…As you get older, you just get more sure of yourself don’t you?*” [Molly].

In this example, Molly is reflecting on how the NVS role may be seen by some as unsettling societal understandings and expectations of veterinarians, meaning that they are no longer seen as “*a vet, this wonderful person that they look up to*”, but “*like the scum of the earth*”. Wilkins (2008; p 3) claims that “*Society expects veterinarians to be involved wherever animals are at risk or are about to be placed at risk*”, yet here is Molly describing the reactions of “*horror*”, when discussing their role within animal research. Elsewhere in the interview, Molly notes that other veterinarians working outside animal research express similar shock and indignation towards their employment as an NVS:

*“It’s pretty similar to the general public, I think. “How could you do that? I could never do that” and yeah, a lack of knowledge of what it involves*” [Molly].

Molly connects these attitudes to a lack of knowledge, pointing to barriers that prevent a shared understanding between those on the inside of the animal research arena and those on the outside, including other veterinarians. This bears similarity with other animal welfare contexts. For example, Hamilton *et al.*’s (2021; p 1116) study of raw milk farmers found perceptions of openness as risky, with opening farms to visitors regarded as potentially fuelling “*unease, disgust or criticism on the part of consumers*”. Similarly, some NVSs hold concerns that their role will be misunderstood and, as will be discussed in the following section, openness can therefore be seen as risky.

On the other hand, participants also discussed the value of informing others about the necessity and value of scientific animal use. Here, Michelle provides a narrative about a local animal rights group who were trying to encourage people to boycott an organisation, because dogs were going to be used in research there:

‘’[…] *But when you actually started talking to her and saying, “Well, do you vaccinate your dogs? How do you think they’ve developed vaccines for dogs? This is why dogs have to be used in some of the research”, she actually started thinking about it a bit more deeply*” [Michelle].

In their interview, Paul makes a similar point:

“*They don’t join it up. I’m not being condescending or patronising, I hope, but I don’t think they really think about how we know that medicines are effective, and medicines are safe. And they might be in theory, very against animals being used in experimentation but then if you say to them, “well, if your child is ill, are you happy for it to have medicines tested on animals or do you want untried, untested?”* [Paul].

This interview data could be analysed in different ways. By referring to obligations towards the health and well-being of family members and pets, the interviewees are making connections with their critics’ care responsibilities, particularly towards legal dependents (and see McGlacken 2021; p 7). However, the same data could also be analysed as a rhetorical move, through which the speaker is minimising the complexity of animal research by constructing it in terms of a harm-benefit framework. Following, Michael and Brown (2004; p 394), this framing potentially neglects lay understandings of animal research which tend to go “*beyond and beneath*” this to “*encompass a series of concerns and views that eventually render those cost-benefit arguments highly spurious*”.

Beyond attempts to educate or inform those who might seek to challenge the idea of animal research and the veterinarian’s role within it, other interview accounts suggest that some veterinarians appreciate the opportunity to be open, not simply regarding animals and their welfare, but also about their own mixed feelings around animal research. For example, Peter reported liking speaking to others:

*“… I’m not very good at lying when folk ask what I do, I’ll say I’m a vet and they’ll say where, and I say “out on contract”. If they ask further questions, I’m quite happy to discuss it and it’s not always justifying it. It’s saying, “Well this is what’s done, I’m against animal testing myself, but while it’s driven by regulations then we have to do it." So yeah, it’s an unavoidable legal process*” [Peter].

In this example, Peter is keen not to be seen as “*always justifying it*” and stresses the legislative necessity of their involvement in a practice they themselves are personally “*against*”. This seemingly conflicting position, being morally opposed to a job that one is performing, highlights the ambivalence that some NVSs may feel in being tasked with jobs that are mandated but which may clash with veterinary professional ethics (and personal morality) to “*ensure the health and welfare of animals committed to my care*” (Royal College of Veterinary Surgeons [RCVS] 2022). It may also reveal the way in which, as predicted by Ashforth *et al.*, those engaging in dirty work can “*simultaneously identify and disidentify with their work*” (Ashforth *et al.* 2007; p 169). Yet, unlike Ashforth *et al.*’s studies where the occupation is seen as ‘dirty’ by others (Ashforth & Kreiner 1999; Ashforth *et al.* 2007), it is the ethical problem of scientific animal use itself that can also create ambivalence for those involved in it. This is neatly summarised by Parker:

“*I don’t think you can be for or against. You’ve got to try to, I think it’s very, very important and it’s absolutely clear, people have a strong voice against animal research* […] *This is the best thing that happened because it forces people to refine, to reduce and to replace, and this is great.* […] *In all of the associations I take part of, I always say the that we’re all working to be unemployed*” [Parker].

In this extract, Parker is partly aligning themselves with public opposition to animal research and the demand for the eventual full replacement of animal models. Hence, rather than seeking to ‘clean up’ the public image of animal research, Parker is reporting that they value open public critique and its associated impact with driving good scientific practice (i.e. the principles of the 3Rs: the Replacement, Reduction, and Refinement of animal use) (Russell & Burch 1959).

Relatedly, Brunt and Weary (2023; p 3) also found that some laboratory veterinarians working in Canada valued transparency as offering “*opportunities for discussion and learning from diverse perspectives*”. Indeed, our UK analysis has echoed this finding. For example, Nadir states: “[…] *What we as a body of lab animal vets do is controversial. In some areas it’s complicated and it takes some explaining*…” [Nadir]. In this example, “*some explaining*” is not about explaining *away* the controversy of animal research and veterinarians’ involvement in it, but instead may be more about a desire to communicate the nuances of the NVS role. Hence, later in the interview, Nadir advocates for finding a “*middle ground*” in understanding scientific uses of animals as having certain scientific value but also as being problematic:

“[…] *those who say that use of animals in research is a busted flush, I think are wrong. But those who say that the use of animals in research is highly problematic are right. So, we have to find the middle ground*” [Nadir].

Overall, this section has explored the different ways in which veterinarians practise or embrace openness around their work. These examples are complex but relate to the idea that openness can provide opportunities to educate others, including other veterinarians. However, the section also demonstrated how some veterinarians seem to value openness not only as a way of communicating the value of animal research and educating about their role, but also as a way of sharing their own ambivalences and reflections on the complexities of scientific animal use. This implies that rather than aiming to *resolve* concerns or controversy, openness agendas should accept the ethical complexity of scientific animal use and the plurality of both public and professional views around it.

### Avoiding openness

Whilst some of the previous interview extracts show an engagement with or appreciation for openness around animal research, other NVSs interviewed expressed discomfort around openness, predicting negative reactions and choosing instead to avoid discussing their work altogether. This might be considered unsurprising, especially given the way in which those in other ‘dirty’ professions may avoid encounters with ‘outsiders’ (Ashforth *et al.* 2007). Indeed, as Obadiah explains in relation to discussing their role, “*I try to avoid as much as I can*”. In their case, even conversations with other veterinarians working in routine general practice were avoided, given the way the latter would react with “*words or these eyebrows and comments. I’m treating animals, not killing them like you*” (*ibid*).

However, such reported avoidance is not necessarily evidence of an individual’s own sense of guilt or shame about their work. Rather, as Arluke (1991; p 316) previously observed in their study of animal research professionals, may be motivated by a desire to avoid ‘long debates’, with an implicit or explicit questioning of their personal morality. This sort of hesitation was articulated by Nando:

“*It depends how I feel, what I know about the people, whether I can be bothered to get into discussion about it. I’ve got no problem defending what I do and describing what I do, but if I think it’s going to lead to awkwardness in a social situation or arguments, I might well duck it*” [Nando].

Another participant similarly describes how the possibility of being forced into a justification of their work sometimes makes them reluctant to get into a conversation on the topic:

“[…] *I don’t really want to get involved in a difficult conversation and have to talk about how I justify what I do and “how could you possibly do that to sweet little furry animals?”, I just don’t want to have the conversation. I’m not particularly worried about any kind of violence or retribution or embarrassment or anything, it’s just too much hassle*” [Max].

Rather than claiming that they are unable to be open, Nando and Max both hint at the labour involved in discussing their work, with such conversations described as being had if *“I can be bothered to get into discussion about it*” [Nando] or sometimes judged as “*just too much hassle*” [Max]. Again, social scientific approaches can help make sense of these accounts. Expanding on Hochschild’s (2003) influential concept of ‘emotional labour’, Ladegaard *et al.* (2021; p 3) define ‘defensive labour’ which involves “*anticipating, managing and neutralizing emotional, physical, or economic workplace threats, where the adversarial consequences primarily fall on the worker, rather than the firm*”. In the case of the NVS, such acts of defensive labour may thus be undertaken to minimise the physical risks of disclosure as well as the labour involved in providing long explanations. Indeed, following Ladegaard, this resistance towards performing openness may be understood as related to the positioning of responsibility for animal use in science. Here, a reluctance to engage may reflect a pushing back of accountability for the *decision* to use animals, which is not made directly by veterinarians, but by researchers. In this case, some NVSs may feel it unfair that they should have to deal with the ‘adversarial consequences’ of animal research, when their main responsibility is to mitigate the impacts of experimental choices on animal welfare.

As well as deliberately avoiding conversations about their work, some participants also reported examples of strategically drip-feeding bits of information about their role, whilst testing the safety of the encounter. As Maddison discusses:

*“*[…] *when you say I’m a vet they picture James Herriot or the small animal vet in a white coat. That’s it, so you’re safe saying I’m a vet. Then I would say I work in an animal health institute. Then normally that’s where the conversation stops because they don’t know what that is. People closer to me, they know exactly what I do and we have interesting discussions at times. It’s very little understood*” [Maddison].

These examples of strategically navigating how information much is revealed were also observed in Arluke’s (1991; p 314) US study and in Brunt and Weary’s (2021; p 4) Canadian study, with the latter finding that managers of animal facilities “*described how they modulated information sharing to shape conversations, explaining that they did so based upon how supportive the person they were speaking with was of using animals in science*”. As Maddison in our study alludes to, the ‘James Herriot’ veterinary identity can be used to strategically hide behind. However, as Obadiah’s experiences of negative comments from other veterinarians discussed earlier indicate, reliance upon this societal image of the veterinary profession may not always work. Indeed, as previously reported by Anderson and Hobson-West (2024), some NVSs themselves make distinctions between ‘real’ veterinary work and the NVS role.

In some cases, participants described a lingering fear of intimidation or violence towards themselves or their families as a justification for avoiding openness. To be clear, such concerns were not reported by all interviewees and in various instances these anxieties were discussed in the past tense. For example, Mia recalls her decision to keep her work secret from her children when they were younger:

“*We did have an incident where we were targeted as a veterinary practice by animal rights and so I was really concerned about them turning up outside the school gates and by inadvertently one of my children saying something in a school debate* […] *that it might have led someone to put two and two together and put them in some sort of danger*” [Mia].

Likewise, Olivette talks about feeling concerned when she first started at her organisation, although these concerns were not wholly located in the past:

“*it’s all happy, jolly at the moment. But when I started at [organisation], we still had the groups of people standing outside with placards and being unpleasant about it. And the chappies who all went to jail are all about to come out again, aren’t they? It’s been a 12-year or 13-year period, and the internet is very open, once your name and your face and maybe even your address is out there, it’s out there for always…So, from my point of view, at work I could see at home, my married name, we don’t appear on the form because I simply just don’t want to attract trouble to my house if I can help it.* […]” [Olivette].

As Olivette suggests here, the “*trouble*” that making visible their involvement in animal research might attract may have consequences that extend beyond the individual and the workplace. These concerns seem to echo those reported two decades ago, with one UK NVS writing in the veterinary journal, *In Practice*, that “*The down side of NVS work includes the security aspect – I am very cautious about telling friends and even family what I do and am aware of the drill to be followed if suspicious packages arrive in the post*” (Anonymous 2004). Later empirical studies also point to the ‘selective openness’, practised by those involved in animal research (Holmberg & Ideland 2010). Likewise, Brunt and Weary (2021; p 7) describe animal research managers practising a “*selective openness*”, “*believing that transparency is good in principle but fearing it in practice*”. In summary, then, policies of openness around animal research may be valued and promoted by institutions and animal research advocacy organisations (UAR 2014), yet perceived as inappropriate or challenging by some individuals within the field.

Finally, given that some Named Veterinarians also work in clinical practice (Anderson & Hobson-West 2022), risk management of ‘outing’ oneself to clients and potentially losing custom was discussed as another factor motivating a careful approach to openness around their work:

“*Yes, I think within my own practice I would worry whether that might affect my client base, whether it might, I suspect if it was widely known I suspect my clients would split into two camps, one who supported me and one who said, “well we don’t like that and we’ll go somewhere else”*” [Oliver].

Such mediation of openness about their role as an NVS may therefore not only be connected to the potential social ramifications, but the financial ones too. Confirming Brunt and Weary’s work, which shows how Attending Veterinarians in Canada identify institutional concerns about reputational risks as barriers to transparency (Brunt & Weary 2023; p 4), this analysis highlights the additional risks that Named Veterinarians may face in enacting openness around their role. Indeed, for veterinarians working in both research and clinical practice, publics may represent both citizens with a stake in animal research and the veterinarian’s role within it, and also clients, to whom veterinarians have particular obligations and financial dependencies.

### Challenging the portrayal of the NVS within openness agendas

Thus far we have discussed the ways in which NVSs practise or avoid openness around their role. However, the analysis also identified some examples of participants articulating a misalignment with the way in which their profession has been portrayed within openness agendas. While less prominent throughout the interviews, this finding is important to acknowledge for the way in which some Named Veterinarians expressed scepticism towards representations of their role. This was exemplified by Max:

“*I don’t tend to talk about my work outside work […] So, my thoughts aren’t derived from conversations with people outside. My impression is that where people think about it at all, they think we’re some kind of referee or guarantor of animal welfare and I think many institutions present us in that way. So, if you go on the web and look at various establishments’ websites they say, “We have vets to look after the welfare of the animals” […] I think it’s a bit duplicitous […] Because that’s not the role that we have been given in the legislation. That’s not why we’re employed or at least it’s not why I’m employed*” [Max].

Such discursive pushback against representations of the NVS as someone who can guarantee the health and well-being of research animals suggests the difficult position Named Veterinarians are sometimes placed in, with polarised societal imaginations of their work in which they are either assisting in animal harm or acting as a “*guarantor of animal welfare*”. With constructions of the NVS as a signifier of animal welfare, openness around their role may be further impeded, obscuring the nuances of the role and any personal and professional ambivalence towards the scientific use of animals.

Indeed, Jennings and Hawkins (2015; p 4) have previously raised issues with representations of Named Veterinarians, contending that:

“*Given the popular mindset that the presence of a vet will ‘make everything alright’ for animals, in our view it is somewhat disingenuous for establishments and other organisations to use the attending vets to try to allay concerns about, or ‘normalise’ animal use*”.

Such concerns may also help to explain why, in the Canadian animal research context, one potential interviewee refused to participate in Brunt and Weary’s study, citing previous experiences which “*made them sceptical and cynical about institutional commitments to transparency*” (Brunt & Weary 2023; p 6).

If our analysis is correct, then the continued linkage of welfare, veterinarians, and the openness agenda could potentially serve to hinder the societal trust that openness agendas may intend to cultivate (Williams & Hobson 2020). In other words, misleading or simplified representations of the NVS role may have negative consequences for interpersonal openness outside of the workplace, requiring the mitigation of misplaced societal expectations. On this point, recalling when they were unable to attend an open day, another participant described how establishments may use the NVS for “*public relations*”:

“*I think the vet’s name is taken in vain quite a lot because I think a lot of establishments and this is no better or worse than any other, they like to be able to say to the public, “We have a veterinary surgeon on 24-hour call”. And this came up, we’ve got an open day coming up* […] *I said, “I’m really sorry, I can’t come this year” and they said, “that’s a shame because the public like to see that we’ve got a vet”, so there is an element here of public relations, having a vet onboard* […]” [Owen].

Owen’s retelling of the event indicates their view that the veterinarian is being used to help establish or sustain public trust, but this is interpreted as taking the vet’s name “*in vain*”. Owen’s candid description of the situation as “*public relations*” also suggests how such attempts to spotlight the presence of the veterinarian in relation to animal welfare may be experienced as problematic by those performing the role.

Once again, concepts from social science can help to make sense of this dynamic. For example, examining corporate representations of care during the COVID-19 pandemic, Chatzidakis and Littler (2022; p 2) identified the practice of ‘carewashing’ which they define as “*communication strategies designed to demonstrate how ‘caring’ a corporation is in ways that commonly obscure that corporation’s actual destructive social and environmental impacts*”. Given the important regulatory role of the NVS in relation to animal care, such gesturing to the presence of the NVS in research establishments as evidence of animal welfare could similarly be analysed as an example of the ‘carewashing’ of animal research. Using this lens, the excerpt from Owen’s interview cited above may reflect a discursive resistance to carewashing, suggesting their disagreement with strategic institutional moves to spotlight practices of care (as embodied by the veterinarian), and the ways this might minimise the harms involved in animal research.

Stepping back, we could also ask to what extent veterinarians are (or are not) able to help construct the image of their own profession. For example, Friese (2019; p 294) observes that both research animals and animal technicians are often “*represented by others but very rarely represent themselves*”. Following our analysis, we can now add that NVSs may also have limited avenues to discuss their relations with the societal and ethical elements of their work. These empirical findings reinforce Carbone’s (2021; p 15) claim that, alongside scientists and animal technicians, veterinarians involved in animal research in the US:

“[….] *are themselves discomfited by the animal harms their research entails, and might welcome challenging questions and suggestions about their work. They want something other than the* “*my side versus the other side” engagement* […] *They want to be able to talk to people they meet about the work they do, both the good and the bad, without fearing vilification*”.

Overall, this section has indicated that some Named Veterinarians may not necessarily support efforts to ‘clean up’ the image of animal research in ways which minimise the welfare harms involved, or the ethical issues raised.

### Animal welfare implications

Our argument has key implications for animal welfare. Firstly, this work prompts us to further consider the implications of framing openness around animal research as a responsibility of those involved, despite differing positions and relations to the decisions behind their use. As our analysis has shown, some Named Vets position themselves within a ‘middle ground’ regarding the issue of animal research, in which the controversy and ethical problems it raises are acknowledged and engaged with, rather than minimised. If openness around animal research is narrowly equated to the idea of debating or defending the necessity, value, or ethical acceptability of scientific animal use, animal welfare professionals such as NVSs and animal technicians, who often frame their role as *advocates* for animals in research facilities (McGlacken *et al.* 2023), may find openness uniquely challenging, being unable to fully articulate their own complex positions within the practice.

In this way, an incapacity for Named Vets to express the nuances of their position may have consequences for the enactment of their role. For instance, if the NVS’s presence is promoted as a ‘guarantee’ of animal welfare, some may feel restricted in how they can advocate for the welfare and interests of laboratory animals publicly, being caught between polarised imaginations of the laboratory space as either devoid of care, or as a space where animal welfare is certified because it falls under the charge of a number of professional roles and trusted figures. By contrast, a healthy culture of openness should be able to accommodate the tensions raised by ways that harm, care, welfare, and ethics materialise within the context of animal research, generating continuous friction that personnel, across their diverse positions, must navigate together. Returning back to Reed’s (2012; p 251) assessment of the potential benefits of openness, openness can only “*lead to benefits for animals, as third parties*” if laboratory personnel, particularly those responsible for animal welfare, can express the complexities and challenges inherent to such roles.

For veterinary practice, our analysis supports the call for more dedicated spaces for Named Veterinarians to discuss the professional and ethical complexities of their work (Millar 2018). Within such spaces, Named Veterinarians may also find it useful to discuss the significant labour that is involved in embracing and practising openness at an interpersonal level. This might also support their own well-being and job satisfaction, helping to prevent burn-out and veterinary attrition, issues that have been much discussed throughout the wider profession (Lovell & Lee 2013; Moses *et al.* 2018; Veterinary Record 2020; Anderson & Hobson-West 2022) and which, ultimately, represent challenges for animal welfare. We also recommend further academic research focused on the veterinary profession to help avoid simplification of veterinary roles. For example, Named Veterinarians have reported grappling with complex ethical issues when based in clinical practice (Anderson & Hobson-West 2024), and veterinarians more generally have signalled a desire for “*better understanding of societal, professional and individual expectations*” of their own role (Armitage-Chan *et al.* 2016; p 5).

Finally, the promotion of an image of veterinarians as ensuring animal welfare may also have implications for public imaginations and expectations of the lives of research animals, potentially shaping how publics relate to animal research and exert civic pressure upon policy and practice. Linking to the societal contexts through which animals become veterinary patients in the broadest sense, accurately representing the lives of animals in research facilities, which includes the nuanced work of laboratory animal care and welfare professionals, is therefore in the interest of publics and research animals themselves.

Veterinary work, across all domains, involves complex negotiations over the health, welfare, and care of animals for which the veterinarian should not be assumed to be responsible for *solving*, but whose work is crucial to mediating. Indeed, Ashall (2022; pp 10–11) describes veterinarians as “*mediators in the complex relationships between humans and animals*” and argues that they are tasked with “*cleansing society of the obligation to feel the unfiltered consequences of their relationships*”. As Hughes (1962; p 93) observed in their seminal essay on the topic, the concept of ‘dirty work’, “*raises the whole problem of the extent to which those pariahs who do the dirty work of society are really acting as agents for the rest of us*”. Therefore, for the promotion and protection of animal welfare, perhaps the more uncomfortable or unpalatable aspects of both animal research and veterinary practice should be studied in greater detail and brought into the open, in order that society as a whole can better share the burden of responsibility for them.

## Conclusion

This paper has drawn upon qualitative in-depth interviews to provide a detailed analysis of how NVSs in the UK discuss their experiences and engagements with openness in animal research. In line with previous studies in this area (Holmberg & Ideland 2010; Brunt & Weary 2021), this paper confirms that openness can be experienced by animal research professionals as complex or difficult. Yet, this analysis goes further to illustrate how Named Veterinarians may face additional layers of challenge, negotiating conflicts between a public and professional identity primarily based around animal care and their role within the research environment which sometimes involves intentional animal harm. In other words, this study moves us beyond narrow ideas of ‘saving animals’, with veterinarians caught up in sometimes contested practices of caring, harming, and killing (Venkat 2021). Furthermore, we have argued that Named Veterinarians find certain representations of their work uncomfortable, with some participants expressing a more complicated relationship with their image as ‘guarantor’ of animal welfare. This analysis therefore has key implications for the kind of openness we want to foster around animal research, whose interests this serves, and how it may impact upon the capacity of NVSs to advocate for animal welfare.

That multiple participants in this study raised challenges of being open about their role as an NVS suggests that a healthy culture of openness around animal research requires a new approach. Continuing to set animal research up as an issue that can be ‘solved’ or a debate to be ‘won’ may obscure the ambivalent relations that professionals such as veterinarians have, not only with their own ethical relationship towards scientific animal use, but also with the way in which their professional role is portrayed by others. Indeed, previous research has argued that this framing distorts the nuanced relations that wider publics have with the topic (McGlacken & Hobson-West 2022; McGlacken 2022).

Hence, this analysis stresses the wider importance of fostering *dialogue* around animal research, which as Hyde and Bineham (2000; p 212) describe, is “*non-polarized discourse*”, being “*not oppositional, but collaborative*” and seeks “*not the ascendance of one perspective over another, but the fusion of all perspectives to enable a larger, more inclusive view, one which allows the tension of disagreement*”. For example, speaking of their work to facilitate dialogue between science and society around the animal welfare of farmed animals, Miele *et al.* (2011; p 116) suggest that “*dialogue can increase trust and respect even if ideological differences remain*”. In the animal research context, this means supporting scientists and animal care professionals to be open, not only regarding their work, but also about the ethical problems, concerns, and critique it may raise. Such a dialogue is critical to developing a culture around scientific animal use which is open not only to sharing information but also to engaging with new understandings of the issues at stake and new possibilities for acting upon them. Indeed, as Brunt and Weary (2023; p 6) conclude in their Canadian study, sharing more information with publics as well as seeking to understand ‘minority opinions’ might “*improve decision-making through the incorporation of social values*” and also “*begin to address value-ladened issues like animal welfare*”.
